# Reliability and concurrent validity of the International Physical Activity Questionnaire short form among pregnant women

**DOI:** 10.1186/s13102-017-0070-4

**Published:** 2017-03-14

**Authors:** Birgitte Sanda, Ingvild Vistad, Lene Annette Hagen Haakstad, Sveinung Berntsen, Linda Reme Sagedal, Hilde Lohne-Seiler, Monica Klungland Torstveit

**Affiliations:** 1 Faculty of Health and Sport Sciences, University of Agder, P.O. Box 422, 4604 Kristiansand, Norway; 2 Department of Obstetrics and Gynaecology, Southern Norway Hospital Trust, Kristiansand, Norway; 3 Norwegian School of Sports Science, Oslo, Norway

**Keywords:** Activity assessment, Activity monitor, Exercise, IPAQ-SF, Physical activity, Pregnancy, Self-reported questionnaire

## Abstract

**Abstract:**

Sanda B, Vistad I, Haakstad LAH, Berntsen S, Sagedal LR, Lohne-Seiler H, Torstveit MK. Reliability and concurrent validity of the International Physical Activity Questionnaire short form among pregnant women.

**Background:**

The International Physical Activity Questionnaire short-form (IPAQ-SF) is frequently used to assess physical activity (PA) level in the general adult population including pregnant women. However, the reliability and validity of the questionnaire in pregnancy is unknown. Therefore, the aims of the present study were to investigate test-retest reliability and concurrent validity of IPAQ-SF among pregnant women, and whether PA is reported differently among those who fulfill (active) vs. do not fulfill (inactive) recommendations of ≥150 min of weekly moderate intensity PA in pregnancy.

**Method:**

Test-retest reliability was examined by answering IPAQ-SF twice, two weeks apart (*n* = 88). To assess validity, IPAQ-SF was compared to the physical activity monitor SenseWear Armband® (SWA) (*n* = 64). The participants wore SWA for 8 consecutive days before answering IPAQ-SF. PA level was reported as time spent in moderate-, vigorous- and moderate-to-vigorous intensity PA (MPA, VPA and MVPA) corresponding to the cut-off points 3–6, >6 and >3 Metabolic Equivalents (METs), respectively.

**Results:**

Test-retest intraclass-correlation of MPA, VPA and MVPA ranged from 0.81-0.84 (95% Confidence Intervals: 0.69,0.90). Comparing time spent performing PA at various intensities; the mean differences and limits of agreement (±1.96 Standard Deviation) from Bland-Altman plots were−84 ± 402 min/week for MPA,−85 ± 452 min/week for MVPA and 26 ± 78 min/week for VPA, illustrating that the total group under-reported MPA by 72% and MVPA by 52%, while VPA was over-reported by 1400%. For the inactive group corresponding numbers were 44 ± 327 min/week for MPA, 52 ± 355 min/week for MVPA and 16 ± 33 min/week for VPA, illustrating that the inactive group over-reported MPA by 13% and MVPA by 49%, while VPA was not detected by SWA, but participants reported 16 min of VPA/week. In contrast, corresponding numbers for the active group were−197 ± 326 min/week for MPA,−205 ± 396 min/week for MVPA and 35 ± 85 min/week for VPA, illustrating that the active group under-reported MPA by 81% and MVPA by 60%, while they over-reported VPA by 975%.

**Conclusion:**

IPAQ-SF had good test-retest reliability, but low to fair concurrent validity for MPA, VPA and MVPA compared to an objective criterion measure among pregnant women. Further, women fulfilling PA guidelines in pregnancy under-reported, while inactive women over-reported PA level.

## Background

Physical activity before and during pregnancy promotes health for both the mother and the unborn child [1–4]. Healthy pregnant women are recommended to be physically active for at least 150 min per week at moderate intensity, or to continue their pre-pregnancy physical activity level if these recommendations are already met [1, 5–7]. However, studies suggest that physical activity recommendations are rarely met, either in the general population [8–10] or among pregnant women [11]. Additionally, physical activity levels tend to decline during pregnancy [12, 13]. Feasible, reliable and valid measures of physical activity before, during and after pregnancy may aid in investigating changes over time, measuring effectiveness of health promotions and interventions and evaluating population trends [14]. Physical activity entails complex behaviour. Identifying the most accurate way to capture total physical activity level is challenging, as different methods have strengths and limitations [15]. A wide range of objective and subjective techniques (including indirect calorimetry, accelerometers, inclinometers, heart rate monitors, multisensors, pedometers, doubly labeled water, diaries, self-reported and interview-administered questionnaires) have been applied to record physical activity in different populations, including pregnant women [16, 17]. In large-scale surveys, self-reported questionnaires are widely used to estimate physical activity level due to their low cost and easy distribution [18–21]. Women who are regularly physically active may have better comprehension of physical activity intensity and duration, and thus may be more accurate in reporting physical activity [22]. There is limited knowledge whether such differentiated perception of physical activity affects the responses in self-report questionnaires.

Short, user-friendly questionnaires of good reliability and validity are preferable to corresponding lengthy ones, if the same information is captured. Further, a questionnaire capable of assessing physical activity levels before, during and after pregnancy would be useful. Most validated self-reported pregnancy-specific questionnaires are lengthy, only for use during pregnancy [23–25], and few are tested for reliability [24]. The International Physical Activity Questionnaire short form (IPAQ-SF) is designed with only 7 questions and is frequently used to assess physical activity level in large-scale population-based studies, including during pregnancy [18–20, 26]. The long version of IPAQ, which assesses physical activity across different domains such as leisure-time, home-life, work and transport, has been validated among pregnant women [27], showing poor correlation between the questionnaire and an accelerometer (0.03 for moderate physical activity (MPA), 0.15 for total physical activity). To our knowledge, the IPAQ-SF has not been tested for reliability or validity in a pregnant population.

Hence, the main aim of the present study was to evaluate the two-week test-retest reliability, as well as the concurrent validity of the last 7-day IPAQ-SF among pregnant women. In addition, it was of interest to investigate possible differences in concurrent validity of IPAQ-SF in women classified as “inactive” or “active”, based on whether or not they fulfilled current physical activity recommendations in pregnancy.

## Methods

### Study design

In the reliability study, the participating women filled in the paper version of IPAQ-SF questionnaire twice with a two-week interval. In the validity study, IPAQ-SF was compared to SenseWear Armband® (SWA). The participants wore the SWA for eight consecutive days, while maintaining their usual daily routines, before answering IPAQ-SF electronically. Further, based on SWA measures, participants were divided into an inactive group, including those who did not fulfill the national physical activity guidelines at 150 min of MPA per week (*n* = 30), and an active group including those who fulfilled the national physical activity guidelines (*n* = 34) [5]. Written consent was obtained from all participants and the Regional Committee for Medical Research Ethics South-east approved both studies (REK reference 2009/429).

### Participants and data collection

#### Reliability study

A convenience sample of 154 pregnant women referred for routine ultrasound to Southern Norway Hospital Trust, Kristiansand, between October 2009 and December 2010 were recruited to participate in the test-retest study. The participants received a written invitation one week before their scheduled routine ultrasound examination, around gestational week 18–20. Those literate in Norwegian were eligible for participation. Eighty-eight participants (57%) completed both questionnaires within four weeks, and were included in the present analysis.

#### Validity study

Participants in the validation study were recruited at the time of routine ultrasound examination at the outpatient clinic at Southern Norway Hospital Trust, Kristiansand (*n* = 108), from local health care centres (*n* = 2) and through advertisement on Facebook (*n* = 8). Eligible participants received oral and written information about the project, along with information on how to wear the SWA and how to get access to the web-based IPAQ-SF. They were also asked to report any non-wear time periods during their wear period. All participants were informed that IPAQ-SF reflected their physical activity level for the past week, and that the intention of the study was not to evaluate their physical activity level, but to compare two physical activity measures. Women between 14–35 weeks of gestation who were literate in Norwegian were eligible for the study, excluding those with allergy to nickel since the SWA contains eight percent nickel. Recruitment was done over two periods, November - December 2013 and May 2014 – January 2015. Of 118 enrolled, complete dataset for analysis was obtained from 64 participants (54%). Data from 54 women were excluded because of technical instrument failures (*n* = 13), missing IPAQ-data (*n* = 3), withdrawn consent (*n* = 3), developing rash (*n* = 3), a late spontaneous abortion (*n* = 1) or based on the IPAQ–SF data cleaning protocol (*n* = 31) (those who answered “don’t know” in one or more of the minute categories in the questionnaire).

### Measures

#### International physical activity questionnaire short-form

IPAQ was originally developed as an instrument for standardized measurement of physical activity behaviours in different populations. Various forms were developed, including a 7-item short “last 7 day” self-reported form (IPAQ-SF) [28]. The reliability and validity of IPAQ have been tested in adult populations [28–30]. IPAQ-SF quantifies physical activity during the last seven days divided into four categories: vigorous intensity, moderate intensity, walking and sitting. In addition to intensity, frequency and duration of physical activity are assessed.

In the category of vigorous intensity, the respondent was asked: *“During the last 7 days, on how many days did you do vigorous physical activities like heavy lifting, digging, aerobics, running or fast bicycling*?” with the possible responses 0 to 7. This was followed by a question of “*How much time did you usually spend doing vigorous physical activities on one of those days?*”*.* The response categories were divided into “*don’t know*”*,* “*10 min*”*,* “*20 min*” and so forth up to “*2 h or more*”. Similar questions follow in the categories moderate intensity physical activity (with examples of carrying light loads, jogging or bicycling at a regular pace, with specification not to include walking) and walking for at least 10 min at a time, followed by the same response alternatives. Finally, in the category “sitting” the respondent was asked “*During the last 7 days, how much time did you spend sitting on a weekday*?” and the response was number of hours. Vigorous physical activity was described as “*activities that take hard physical effort and make you breath much harder than normal*”*.* Moderate physical activity was described as “*activities that take moderate physical effort and make you breath somewhat harder than normal*” (www.ipaq.ki.se)*.*

Responses were scored according to the IPAQ-protocol (www.ipaq.ki.se) after summation of the duration (in minutes) and frequency (in days) of the different physical activity intensities; MPA, vigorous physical activity (VPA), and moderate to vigorous physical activity (MVPA).

#### Sensewear ® armband monitor

SWA (BodyMedia Inc, Pittsburgh, PA, US) was used as an objective comparison measure of physical activity level. The SWA is a multisensory device that contains a 3-axis- (SWA Mini) or 2-axis- (SWA Pro_3_) accelerometer and registers galvanic skin response, skin temperature, heat flux and collects minute-by-minute data. SWA is worn on the upper arm (left arm for SWA Mini and right arm for SWA Pro_3_) on the triceps and at midhumerus point. The predecessor, SWA Pro_2_, has been validated in pregnancy against indirect calorimetry for conditioning exercises, showing that the SWA Pro_2_ underestimated energy expenditure by nine percent [31]. The newer model, SWA Mini, has also been validated in pregnancy against indirect calorimetry, but only for activities of daily living, showing that SWA overestimated energy expenditure in all activities except inclined walking, by a mean of 27.7% and 35.6% depending on algorithm used [32]. Both SWA Mini and SWA Pro_3_ have shown significant agreement with doubly labeled water in measuring total energy expenditure in adults [33]. Nine (14%) of the 64 participants in the present study used the older version SWA Pro_3_ during a period when SWA Mini was not available.

Data were downloaded and analysed using appropriate software (SenseWear Professional Research Software; BodyMedia Inc, Pittsburgh, PA, US software 7.0.0.2378 (Mini), 6.1.0.1528 (Pro_3_)) according to manufacturer. Further, the data were computed into 10-min epochs of MPA, MVPA and VPA, for comparison with IPAQ-SF, with the following cut-off points: 3–6, >3 and >6 Metabolic Equivalents (METs), respectively, corresponding to the compendium-based MET intensities [34]. A day of recording was valid if the participant wore the SWA for at least 19.2 h, i.e. 80% of a 24-h sampling period [35]. A measurement time-frame of at least four consecutive valid days was required in order for an SWA recording to be included in the analysis [36].

Baseline data as socio-demographic variables and weight were self-reported through questionnaires and consent forms.

### Statistical analyses

All data collected were analysed using the statistical software package IBM SPSS Statistics version 22.0 (IBM Corp., Somers, NY, USA). Background variables are presented as median with min-max values, frequencies, or percentages. A two-way mixed, single measure, parametric intraclass correlation (ICC (3.1)) was performed evaluating the extent of agreement of IPAQ-SF in the test-retest analysis. An ICC ≥ 0.70 was considered acceptable [14]. Level of significance was set to five percent. Bland-Altman plots with limits of agreements (mean difference ±1.96 Standard Deviation) are presented as level of agreement between IPAQ-SF and SWA [37]. Spearman correlation coefficient was used for correlations between IPAQ-SF and SWA. A correlation coefficient ≥ 0.50 was considered acceptable, as proposed by van Poppel et al. [14]. Median with interquartile range and logarithmic transformation were used in the analysis of time spent in the respective intensities due to skewed data.

With respect to sample size, no power calculations were done in the reliability study; the number of participants included was considered to be acceptable based on comparable published studies [24, 28, 38]. In the validity study, posteriori sample size power calculation was performed (level of significance: five percent), showing a power of 83% using G*power 3.1.9.2.

## Results

Baseline characteristics for participants in both the reliability and validity studies are shown in Table 1.

**Table 1:** Baseline characteristics of participants in the reliability and validity studies

	Reliability study *n* = 88	Validity study *n* = 64
Variable	Median (min-max)	
Age at inclusion (years)	28 (20–42)	30 (22–44)
Gestational week at inclusion	19 (16–31)	21 (16–35)
Pre-pregnancy weight (kg)	64 (51–103)	63 (50–112)
Pre-pregnancy BMI (kg/m^2^)	22.6 (17.9–38.3)	22.3 (19.5–43.2)
	*n* (%)	
Educational level ≥ 4 years college/university	28 (31.8)	29 (45.3)
Occupation Employed outside home Long-term sick leave	78 (88.6) 1 (1.1)	55 (85.9) 0
Household income (NOK) ≤ 400,000 401,000–700,000 > 700,000 Wish not to answer	12 (13.6) 33 (37.5) 38 (43.2) 5 (5.7)	20 (31.0) 14 (22.0) 30 (47.0) **-**
Evaluation of own health Good/very good	78 (88.6)	59 (92.2)
Marital status Married/cohabitant/partner Single	86 (97.7) 1 (1.1)	61 (95.3) 2 (3.1)
Tobacco habits Non-smoker Smoked pre-pregnancy Daily smoker Daily snuff	67 (76.1) 19 (21.6) 2 (2.3) 0 (0)	48 (75.0) 13 (20.3) 3 (4.7) 2 (3.1)

The test-retest reliability of IPAQ-SF presented as ICC was 0.81-0.84 for MPA, MVPA and VPA (Table 2).

**Table 2:** Test (test 1) and retest (test 2) of physical activity measures and reliability coefficient of IPAQ-SF, *n* = 88

PA intensity	Test 1 PA measures min/week Mean (SD)	Test 2 PA measures min/week Mean (SD)	ICC (95%CI)
MPA	72.9 (141.3)	78.0 (132.4)	0.81 (0.71–0.88) (*p <* 0.001)
VPA	28.1 (61.3)	26.7 (69.9)	0.84 (0.74–0.90) (*p <* 0.001)
MVPA	95.5 (151.5)	107.6 (167.6)	0.81 (0.69–0.89) (*p <* 0.001)

*PA*: physical activity

*SD*: standard deviation

*ICC*: intraclass correlation coefficient

*MPA*: moderate physical activity

*VPA*: vigorous physical activity

*MVPA*: moderate to vigorous physical activity

**Fig. 1:**
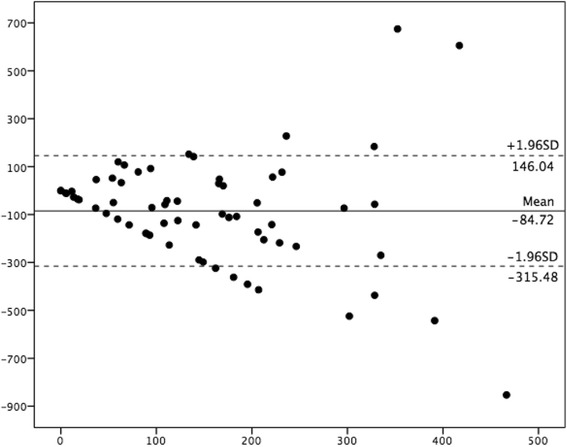
Bland-Altman plot depicting MVPA level over the past week for the total group, *n* = 64 X-axis represent average of IPAQ and SWA (minutes), Y-axis represents IPAQ minus SWA (minutes). (^**______**^) mean difference between the two methods, (− − −) limits of agreement (1.96SD)

**Fig. 2:**
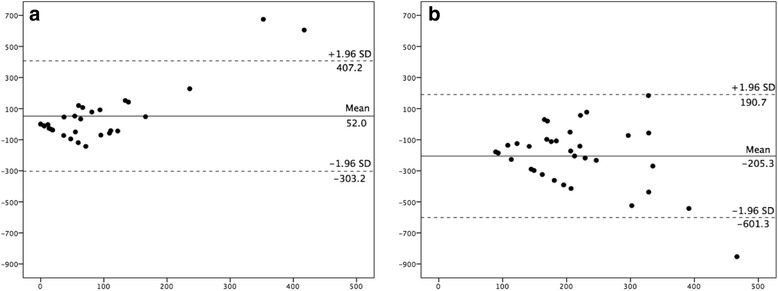
Bland-Altman plot depicting mean difference for MVPA level over the past week. **a** inactive group (*n* = 30) and **b** active group (*n* = 34). X-axis represent average of IPAQ and SWA (minutes), Y-axis represents IPAQ minus SWA (minutes). (^______^) mean difference between the two methods, (− − −) limits of agreement (1.96SD)

Each participant wore the SWA for a mean of 6.7 (5–8) days, with mean on-body time of 23.6 h daily. Comparing time spent performing physical activity at the various intensities, the mean differences and limits of agreement from Bland-Altman plots were −84 ± 402 min/week for MPA, −85 ± 452 min/week for MVPA and 26 ± 78 min/week for VPA (Fig. 1). This illustrates that IPAQ-SF under-reported MPA by 72% and MVPA by 52%, while VPA was over-reported by 1400% compared to SWA for the total group. Further, when participants were divided into two groups based on physical activity level, the mean differences and limits of agreement from Bland-Altman plots for the inactive group were 44 ± 327 min/week for MPA, 52 ± 355 min/week for MVPA and 16 ± 33 min/week for VPA. This illustrates that the inactive group over-reported MPA by 13% and MVPA by 49%; while VPA was not detected by SWA, but the participants reported 16 min of VPA/week in IPAQ-SF. Corresponding numbers for the active group were −197 ± 326 min/week for MPA, −205 ± 396 min/week for MVPA and 35 ± 85 min/week for VPA (Fig. 2), illustrating that the active group under-reported MPA by 81% and MVPA by 60%, while they over-reported VPA by 975%.

Differences between the two measures (IPAQ-SF and SWA) in the different physical activity intensities and their correlations are shown in Table 3. Significant correlations were found in time spent in VPA for the whole sample (*τ* = 0.39, *p* = 0.002), MPA for the inactive group (*τ* = 0.38, *p* = 0.037) and VPA for the active group (*τ* = 0.42, *p* = 0.013).

**Table 3:** The correlation between self-reported time (IPAQ-SF) spent on three different physical activity intensities with measures from SWA, for total-, inactive- and active group, using Spearman correlation coefficient (SCC) (*n* = 64)

Intensity	IPAQ-SF	SWA	
Total group	Median (Q1,Q3)/ *ln mean (SD*) min/week	Median (Q1,Q3)/ *ln mean (SD*) min/week	*τ* (*p*)
MPA	40 (0,120)/*2.8 (2.3*)	139 (47,223)/*4.5 (1.5*)	0.08 (*p* = 0.536)
VPA	28 (42) ^¶^/*1.5 (2.1*)	2 (9) ^¶^/*0.2 (0.9*)	**0.39 (** ***p*** **= 0.002)**
MVPA	80 (0,148)/*3.2 (2.4*)	168 (51,293)/*4.7 (1.5*)	0.14 (*p* = 0.280)
Inactive group (MVPA measured with SWA < 150 min/week) *n* = 30			
MPA	50 (0,125)/*2.8 (2.4*)	44 (14,96)/*3.4 (1.5*)	**0.38** (***p*** **= 0.037)**
VPA	16 (33) ^¶^/*1.0* (*1.8*)	0/*0.0*	-
MVPA	70 (0,125)/*3.0* (*2.5*)	47 (14,120)/*3.6* (*1.5*)	0.25 (*p* = 0.186)
Active group (MVPA measured with SWA ≥150 min/week) *n* = 34			
MPA	40 (0,120)/*2.9* (*2.2*)	210(175,319)/*5.5* (*0.4*)	−0.02 (*p* = 0.905)
VPA	39 (47) ^¶^/*2.1* (*2.2*)	4 (12) ^¶^/*0.4* (*1.1*)	**0.42** (***p*** **= 0.013)** ^**a**^
MVPA	115 (0,180)/*3.4* (*2.3*)	290(193,362)/*5.7* (*1.1*)	−0.06 (*p* = 0.726)

^¶^ Presented as mean min/week (SD) and not as median, due to median and IQR value zero for the skewed data

*IPAQ-SF*: International Physical Activity Questionnaire short form

*SWA*: SenseWear Armband

*Q1*: First quartile, 25% of scores has value lower than Q1

*Q3*: Third quartile, 75% of scores has value lower than Q3

*ln*: natural logarithm

*SD*: standard deviation

*τ* = Spearman correlation coefficient, SCC

*p* = level of significance

*MPA*: moderate physical activity

*VPA*: vigorous physical activity

*MVPA*: moderate to vigorous physical activity

## Discussion

Reliability between test and retest for MPA, VPA and MVPA using IPAQ-SF was good. With respect to validity, comparison of IPAQ-SF estimations of MPA, VPA and MVPA with the reference method SWA showed, however, limited agreement. Physical activity level was under-reported using IPAQ-SF for the total group, in contrast to most self-reporting questionnaires, used both in general and in pregnancy [29, 39]. Interestingly, our results suggested that physically active pregnant women tended to under-report, while inactive pregnant women tended to over-report their physical activity level using IPAQ-SF. This indicates that self-reported estimation of physical activity varies by physical activity level.

### Reliability

We found somewhat higher ICC (0.81–0.84) of IPAQ-SF compared to previous studies investigating test-retest of physical activity questionnaires, where median reliability coefficients varied from 0.62 – 0.76 [40]. Furthermore, the present study demonstrated higher test-retest reliability compared to the initial test-retest of IPAQ-SF conducted among adults in 12 countries (pooled Spearman *τ* 0.76) [28, 40], as well as similar reliability compared to another pregnancy-specific self-reported questionnaire (0.78–0.83) [24] and interview-based questionnaires (0.81–0.84) [38, 41]. Reliability was highest for VPA (0.84), which may be explained by the often planned nature of these activities, making them easier to recall.

To achieve level one of evidence for reliability it is suggested that the time frame between the two questionnaires should be short enough not to change physical activity level, while long enough to prevent recall [14]. The time frame of mean 2.5 weeks, appropriate sample size (>50) and analysis (ICC), as well as good correlation (>0.70) between MPA, MVPA and VPA in the present study supports achievement of level one evidence of reliability, according to points raised by Van Poppel et al. [14]. Though the present study lacks measure of responsiveness, the high correlation coefficients reflect good consistency, which may give IPAQ-SF some value in repeated measures and ability to monitor change in physical activity level over time, as well as ability to compare physical activity levels before, during and after pregnancy.

### Validity

Correlation coefficients for MPA (*τ =* 0.08, *p* = 0.536), VPA (*τ* = 0.39, *p* = 0.002) and MVPA (*τ* = 0.14, *p* = 0.280) in the present study were in accordance with correlation coefficients reported in a review comprising 23 previous studies using IPAQ-SF (between −0.09–0.38 for MPA,−0.18–0.47 for VPA and 0.15 for MVPA) [29]. In addition, the present results are in line with other pregnancy-specific physical activity questionnaires that have been validated against a physical activity monitor, with correlation coefficients between 0.08–0.59 for MPA, VPA and total physical activity for self-reported questionnaires [23–25], and between 0.06–0.59 for MPA, VPA, MVPA and total activity for interview-administered questionnaires [38, 41]). In a systematic review on measurement properties for physical activity questionnaires in adults, Van Poppel et al. (2010) suggested a correlation cut off point >0.50 as sufficient for level 1 evidence of validation when compared to an accelerometer [14]. Few physical activity questionnaires for pregnant women report correlation values >0.50. To our knowledge, a self-reported questionnaire validated by Haakstad et al. [23] and an interview-administered questionnaire validated by Schmidt et al. [38] were the only two studies reporting correlation values >0.50, and only for VPA and total activity/sports-exercise respectively. However, comparison to other studies should be done with caution as studies differ in methodologies that are used, including measurement methods, statistical analysis and cut-off points [14, 41]. These differences might also partly explain the variation in results between studies, in addition to assessment in different trimesters when it concerns pregnancy. According to a systematic review comprising 148 studies, large variations of under- and over-reporting of physical activity level are found ranging from −100% to 4024%, with an average over-report of 138% [39]. Our findings, that physical activity categorized as MPA and MVPA was under-reported by > 52%, are similar to results reported in a Swedish IPAQ-SF validation study using MTI Actigraph as criterion measurement, where MPA and VPA were under-reported by 49% and 31%, respectively, among the female participants (*n* = 98) [42]. Another validation study of a pregnancy-specific questionnaire conducted in Norway, using ActiReg® system as criterion measure, also found that MPA was under-reported, although not to such extent as in the present study (only 16%, MPA *τ* = 0.15, *p* = 0.183) [23].

Pregnancy is associated with large physiological changes in cardiovascular, respiratory, hematologic and metabolic responses, leading to increased heart rate, respiration, resting metabolic rate and absolute energy cost, [43, 44]. These changes in relation with IPAQ’s guidelines of moderate and vigorous intensity (www.ipaq.ki.se) may explain the poor correlation between the two methods included in the present study. The physiological changes may alter the perception of intensity level with respect to physical activity and exercise. The wide limits of agreement of MPA (−84 ± 402) and MVPA (−85 ± 452) for the total group in the present study may indicate that IPAQ-SF does not assess these intensities accurately on an individual level in pregnancy. Further, as significant correlation between IPAQ-SF and SWA was seen only for VPA for our total group, these results indicate that IPAQ-SF alone may have limited value in assessment of physical activity level among pregnant women, especially if use of only one measurement point. The proven reliability of IPAQ-SF may make it suited for repeated measurements of physical activity level over time in study participants.

When dividing our total group based on those fulfilling the national physical activity guidelines or not, the active group (*n* = 34) under-reported their MPA and MVPA with almost three hours/week. In contrast, the women in the inactive group over-reported MPA and MVPA by six (13%) and 23 (49%) minutes/week respectively, which is lower than most findings from previous validation-studies of IPAQ-SF (36–173%) [29]. Our findings are similar to those reported by Shook et al. [45], which demonstrated differences in self-reported physical activity level based on fitness level in the general adult population [45]. In another recently published study using a pregnancy-specific questionnaire [22], self-reported physical activity levels were over-reported among both active and inactive participants. Few studies have, however, focused on possible differences between those defined as physically active and physically inactive. In the present study the degree of under-reporting of MPA and MVPA by the active group was substantially larger than the corresponding over-reporting by the inactive group, resulting in considerable impact on the result for the whole group. Finally, similar to previous studies [30, 45], we found VPA level over-reported in both the inactive and the active group.

There may be several reasons for discrepancies in self-reporting of physical activity level among active versus inactive pregnant women. Perception and tolerance of intensity in a given activity may be different. Due to the physiological changes in pregnancy, as decreased pulmonary reserve, increased cardiac output and systemic vasodilation, the inactive women may have experienced heavier breathing at a lower activity level and classified intensity as moderate in accordance to questionnaire guidelines, while the SWA may only have registered it as light intensity. On the other hand, the active women may have used the physiological responses before pregnancy as reference and thereby felt insufficiently active, which might have led to under-reporting of MPA and MVPA. A Canadian study conducted in 129 adults (*n* = 90 females) highlights the difficulties in selecting the proper physical activity intensity; most participants underestimated MPA and VPA and, when instructed, only 24% walked at a moderate to vigorous pace, while the majority actually walked at light intensity [46]. In addition, in the present study, active women might have spent more time walking compared to the inactive women, captured by the SWA as moderate intensity activity, but according to IPAQ instructions, walking should not be included in the moderate intensity category. The SWA registered significantly more steps among the active- compared to the inactive group (58616 steps vs. 45424 steps/week, *p* = 0.005, data not shown), although quantifying steps does not include aspects such as intensity.

The significant correlation between IPAQ-SF and SWA seen for MPA for the inactive group may suggest that IPAQ-SF may be of some value to assess MPA for inactive pregnant women. Further, when we removed the three outliers seen in the Bland-Altman plot in Fig. 1, we found an association between IPAQ and SWA in assessing MPA and MVPA for the inactive group, with the mean differences and limits of agreement from Bland-Altman plots being 4 ± 138 and 10 ± 172 min/week, respectively (data not shown). However, removing outliers did not change the results significantly for the total or for the active group.

A wide range of self-reported physical activity questionnaires are available, though reviews have shown that it is difficult to point out some that are superior to other [14, 40, 47–49], including those specific to pregnant populations [50]. Accordingly, dose–response relationships between self-reported physical activity level and pregnancy outcomes remain difficult to establish [51, 52]. Though we found limited validation of IPAQ-SF when assessing MPA, VPA and MVPA in pregnancy, only a small number of other self-reported physical activity questionnaires available for use in pregnancy possess overall good validity for measuring different physical activity intensities [23–25]. Therefore, IPAQ-SF’s advantage of brevity and its ability to assess physical activity level preconception, during pregnancy and postpartum, as well as later in life, are of great value. This is especially relevant as the importance of initiating lifestyle changes pre-pregnancy is increasingly recognized [53–55]. Additionally, IPAQ-SF’s good test-retest reliability in this study and for the general adult population [28] supports use of repeated measures.

### Strengths and limitations

Strengths of the present study included an acceptable sample sizes [14], and that all data were cleaned and analysed according to the IPAQ protocol. Another strength is the use of the objective physical activity monitor SWA that combines information about different signals and captures movements. SWA is sensitive for several activities, from sedentary behaviour and sleeping to vigorous physical activity [56]. Furthermore, the SWA is small, light and wireless and localized on the upper arm, a convenient location especially in pregnancy, compared to other activity monitors worn at the waist or hip. In addition, compliance with the SWA was high (mean wear 6.7 days, 98% on-body).

A limitation of SWA is that it must be removed when in contact with water and that it contains eight percent nickel, which may cause skin reactions. SWA has also, like accelerometers [57, 58], been shown to have difficulties in registering inclined walking, rowing and cycling [32, 59, 60]. Another possible limitation of the present study is that we included two different versions of SWA.

Another limitation is that we cannot report responsiveness of IPAQ-SF in the present study [14]; in the test-retest study there is a lack of an objective comparison, while in the validation study there is a lack of two self-report measures.

Characteristics of the women in both our studies, such as age, marital status, household income and smoking habits, are similar to those reported in the largest cohort study conducted on pregnant Norwegian women (the Norwegian Mother and Child Cohort Study) as well as to the general female population of reproductive age in Norway [54, 61, 62]. The majority of women in our studies (61.5% in the reliability study and 75% in the validation study) had higher education (college/university education) which also concurs with what was reported in the Norwegian Mother and Child Cohort Study (59.5%) [54]. A large proportion of the general female population in Norway also has higher education (27.6 - 58.0%, age interval 20–39 years) [63], although somewhat lower than what was observed in the validation study. In the reliability study, 27% of the participants were overweight/obese, which is similar to the prevalence found in the general female adult population in Norway (23% with BMI ≥ 27 kg/m^2^) [64] and in participants in the Norwegian Mother and Child Cohort Study (32.8% with BMI ≥ 25 kg/m^2^) [54]. In the validation study, however, only 10.9% of the participants were overweight or obese. Based on these characteristics, the participants in the reliability study seem to be representative of both the pregnant population and the general female population of Norway, while the participants in the validation study were somewhat slimmer and a slightly larger proportion had higher education. However, as this study aims to test-retest a questionnaire and to compare two measurement methods within the same subject, we maintain our assumption that motivated participants compliant to the planned investigations can provide relevant data for a methodical study. Further, as the two studies aimed to assess measurement properties of IPAQ-SF within each subject, one could argue that the results might have been similar in a random sample from the pregnant population [25]. Additionally, IPAQ has been tested among adults both in developed and developing countries and demonstrated similar results [28].

We have no information regarding non-responders. However, there were no significant differences in socio-demographic variables when comparing those included (*n* = 88 in reliability study, *n* = 64 in validation study) with those excluded from the analysis in the two studies (*n* = 18 in the reliability study and *n* = 31 in the validation study), except for 92% of included women being fully employed outside home in the reliability study compared to 100% of the excluded women (*p* = 0.019).

## Conclusion

IPAQ-SF showed good test-retest reliability, but limited concurrent validity when compared with a sensory-based physical activity monitor in pregnant women.

IPAQ-SF under-reported time spent in MPA and MVPA by > 52% and over-reported VPA by 1400%. The participants’ physical activity level affected the agreement between the questionnaire and the physical activity monitor. Stratifying on whether women fulfilled or did not fulfill physical activity recommendations, the active women under-reported MVPA by 60%, while the inactive women over-reported MVPA by 49%. These findings suggest that participant’s physical activity level should be taken into account when self-reported evaluation of physical activity is done. Physical activity questionnaires are valuable, especially in large-scale population based surveys. Until a questionnaire with improved validity has been developed, objective methods should supplement self-report measures, when possible, in studies investigating physical activity and pregnancy outcome.

## Abbreviations

IPAQ-SF: International Physical Activity Questionnaire Short Form
MPA: Moderate physical activity
MVPA: Moderate and vigorous physical activity
SWA: SenseWear Armband
VPA: Vigorous physical activity
